# Synergistic co-evolution of rhizosphere bacteria in response to acidification amelioration strategies: impacts on the alleviation of tobacco wilt and underlying mechanisms

**DOI:** 10.3389/fmicb.2024.1448950

**Published:** 2024-10-01

**Authors:** Zhang Bian-hong, Tang Li-na, Li Ri-kun, Pan Rui-xin, You Lin-dong, Chen Xiao-yan, Yang Kai-wen, Lin Wen-xiong, Huang Jin-wen

**Affiliations:** ^1^Key Laboratory for Genetics Breeding and Multiple Utilization of Crops, Ministry of Education/College of Agriculture, Fujian Agriculture and Forestry University, Fuzhou, Fujian, China; ^2^Key Laboratory of Crop Ecology and Molecular Physiology (Fujian Agriculture and Forestry University), Fujian Province University, Fuzhou, Fujian, China; ^3^Tobacco Science Research Institute of Fujian Tobacco Monopoly Bureau, Fuzhou, Fujian, China; ^4^Ganzhou Tobacco Company Shicheng Branch, Ganzhou, Jiangxi, China; ^5^College of JunCao Science and Ecology, Fujian Agriculture and Forestry University, Fuzhou, China

**Keywords:** bacterial wilt, acidification amelioration, tobacco, rhizosphere bacteria, synergistic co-evolution

## Abstract

Soil acidification represents a severe threat to tobacco cultivation regions in South China, exacerbating bacterial wilt caused by *Ralstonia solanacearum*. The comprehension of the underlying mechanisms that facilitate the restoration of rhizosphere microbial communities in “healthy soils” is imperative for ecologically managing tobacco bacterial wilt. This study focuses on acidified tobacco soils that have been subjected to continuous cultivation for 20 years. The experimental treatments included lime (L), biochar (B), and a combination of lime and biochar (L+B), in addition to a control group (CK). Utilizing rhizosphere biology and niche theory, we assessed disease suppression effects, changes in soil properties, and the co-evolution of the rhizosphere bacterial community. Each treatment significantly reduced tobacco bacterial wilt by 16.67% to 20.14% compared to the control group (CK) (*p* < 0.05) and increased yield by 7.86% to 27.46% (*p* < 0.05). The biochar treatment (B) proved to be the most effective, followed by the lime-biochar combination (L+B). The key factors controlling wilt were identified through random forest regression analysis as an increase in soil pH and exchangeable bases, along with a decrease in exchangeable acidity. However, lime treatment alone led to an increase in soil bulk density and a decrease in available nutrients, whereas both biochar and lime-biochar treatments significantly improved these parameters (*p* < 0.05). No significant correlation was found between the abundance of *Ralstonia* and wilt incidence. Nonetheless, all treatments significantly expanded the ecological niche breadth and average variation degree (AVD), enhanced positive interactions and cohesion within the community, and intensified negative interactions involving *Ralstonia*. This study suggests that optimizing community niches and enhancing pathogen antagonism are key mechanisms for mitigating tobacco wilt in acidified soils. It recommends using lime-biochar mixtures as soil amendments due to their potential ecological and economic benefits. This study offers valuable insights for disease control strategies and presents a novel perspective for research on *Solanaceous* crops.

## Introduction

Soil-borne bacterial wilt caused by *Ralstonia solanacearum* has led to devastating losses in the yield and profitability of solanaceous crops such as tobacco (*Nicotiana tabacum* L.), potato (*Solanum tuberosum* L.), and tomato (*Solanum lycopersicum* L.) worldwide (Coll and Valls, 2013; Wen et al., 2023; Msabila et al., 2024). Wilt is difficult to control, and its occurrence can result in significant reductions in crop yield and quality, or even total crop failure, hence it is often referred to as “plant cancer” (Yuliar and Toyota, 2015). Tobacco, being one of the most significant economic crops globally, provides substantial employment opportunities in many countries, particularly in developing nations where the tobacco industry is a primary source of income for numerous farmers and laborers. In recent years, soil acidification in tobacco-growing regions of southern China has been exacerbated by factors such as continuous monoculture, environmental pollution, and the application of the use of acidic fertilizers. The rapid proliferation of *R. solanacearum* has resulted in an escalating outbreak of bacterial wilt in these regions, leading to a continuous decline in tobacco yield and quality (Li et al., 2017).

The current measures employed in tobacco production primarily revolve around the development of disease-resistant varieties and the utilization of chemical products for effective control. The disease resistance of the currently selected varieties, however, is not robust enough, rendering them vulnerable to bacterial wilt. The prolonged utilization of chemical agents also poses risks to environmental pollution, pesticide toxicity, and the emergence of pathogen resistance (Meng et al., 2024). The exploration of green control technologies is imperative, necessitating urgent attention. Recent studies have increasingly demonstrated that the occurrence of bacterial wilt is closely associated with imbalances in the microbial community structure within the rhizosphere soil and alterations in rhizosphere microbial diversity. Previous research indicates a negative correlation between higher abundance of beneficial microorganisms in the soil and disease incidence (Dordas, 2008; Dignam et al., 2019; Najeeb and Li, 2023). Consequently, fostering a healthy soil environment and promoting diverse microbial communities are considered highly effective ecological approaches for managing soil-borne diseases. The composition of soil microbial communities is significantly influenced by soil environmental conditions, with soil pH being widely recognized as the most influential predictor, commonly referred to as a key determinant of microbial community structure.

The diversity and stability of bacterial communities are strongly influenced by soil pH (Lauber et al., 2009; Zhalnina et al., 2015). The research findings indicate a significant negative correlation between soil pH and the incidence of bacterial wilt within the pH range of 4.5–6.5 (Yang et al., 2017). This suggests that optimizing soil pH is a fundamental and pivotal strategy for controlling the spread of bacterial wilt. Consequently, there has been a growing interest among researchers in elevating pH levels to mitigate the potential risk associated with bacterial wilt. Lime is a classic soil acidification amendment in agricultural production, and its use is particularly widespread in southern China. Studies have shown that lime application not only increases soil pH but also effectively controls soil-borne diseases such as root rot and fusarium wilt. The key to disease suppression is often attributed to changes in the microbial community, such as reduced pathogen abundance and enhanced microbial community functionality (Chen et al., 2022; Deng et al., 2024). Shen et al. (2018) also found that lime application significantly reduced the incidence of tobacco bacterial wilt, attributing this effect to changes in the rhizosphere microbial community, including decreased abundance of the wilt pathogen and recruitment of disease-suppressive microbes such as *Saccharibacteria* and *Aeromicrobium*. However, the potential long-term risks associated with lime application on soil environments cannot be ignored. Bolo et al. (2021) and Ding et al. (2023) have observed that continuous application of lime may suppress the activity of nutrient-cycling microorganisms, potentially leading to soil “re-acidification.” Furthermore, Liu et al. (2023) revealed that while lime significantly reduced the incidence of tobacco bacterial wilt, it also had a notable negative impact on the diversity of rhizosphere soil bacterial communities. Therefore, there is an urgent need to explore alternative soil improvement measures.

In this context, biochar has increasingly attracted attention as a novel soil amendment due to its characteristics such as enhancing soil fertility, improving physicochemical properties, and optimizing the microbial habitat (Lehmann et al., 2011; Blanco-Canqui, 2017). Recent studies have shown that biochar can adsorb toxic substances and harmful compounds (Kumari et al., 2024). The research conducted by Li et al. (2022) on tobacco plants has demonstrated the effective reduction of bacterial wilt incidence, enhancement of rhizosphere microbial diversity, and decrease in the abundance of the wilt pathogen through biochar application. Our preliminary research also indicates that the primary reason for the reduced incidence of bacterial wilt with biochar application is the improvement in microbial community structure, reduction in wilt pathogen abundance, and increased abundance of beneficial bacteria such as *Bacillus* and *Sphingomonas* (Pan et al., 2024). However, the high cost of biochar remains a significant limitation for its widespread use in agriculture. Despite advancements in disease management through soil improvement strategies and the identification of the close relationship between disease suppression mechanisms and rhizosphere microbial communities, practical implementation in agriculture faces numerous challenges that significantly impede widespread adoption. Therefore, to develop appropriate, efficient, and cost-effective measures and products for the control of tobacco bacterial wilt, it is imperative to investigate the mechanisms underlying rhizosphere microbial evolution during disease development and suppression. Furthermore, researchers emphasize the necessity of deepening our understanding of the mechanisms and co-occurrence patterns of bacterial communities involved in disease suppression to establish a sustainable disease management system. A comprehensive understanding of microbial assembly mechanisms and co-occurrence patterns is crucial for the development of more effective management tools, disease prediction models, and control strategies based on microbial mechanisms (Mallon et al., 2015; Batista et al., 2024).

Therefore, we hypothesize that although different strategies for soil acidification amendment may exhibit variations in their impact on disease suppression in both soil and plants, the fundamental abiotic and biotic mechanisms underlying disease suppression remain fundamentally similar. The critical biotic mechanisms primarily arise from alterations in the process of microbial community assembly and microbial interactions. Accordingly, this research focuses on soils acidified by long-term tobacco monoculture, comparing the efficacy of various amendment strategies in suppressing bacterial wilt and exploring the changes in soil physicochemical properties and the co-evolutionary patterns of rhizosphere bacterial communities during disease suppression. The aim is to elucidate the mechanisms for restoring microbial community equilibrium under “healthy soil” conditions and to provide a scientific basis for establishing sustainable ecological control mechanisms for tobacco bacterial wilt. The findings are expected to offer new theoretical and methodological insights for managing bacterial wilt in other *Solanaceous* crops.

## Materials and methods

### Experimental design

The Fujian tobacco variety “Yunyan 87,” provided by the Tobacco Research Institute of Yunnan Province and the Longyan Tobacco Company of Fujian Province, was utilized in this study. Field trials were conducted over 2 years from 2021 to 2022 at the Fujian Tobacco Agricultural Research Base in Huanxi Town, Jin’an District, Fuzhou City, Fujian Province, China (119°36′86″E, 26°17′33″N). The experimental soil was a red soil paddy field under continuous tobacco-rice rotation for 20 years. From January to August 2021, a randomized block design was implemented, comprising three treatments: a control group without any soil amendments (CK), the application of 1,500 kg·ha^−1^ lime (L), and the application of 30 t·ha^−1^ biochar (B). Each treatment had three replicates, resulting in a total of nine plots with dimensions of 144 m^2^ (24 m × 6 m). From January to August 2022, an additional treatment was introduced: the combined application of 750 kg·ha^−1^ lime and 15 t·ha^−1^ biochar (L + B). This led to four treatments, each with three replicates, totaling 12 plots.

One month before tobacco planting, whole top soil layer amendments were conducted, followed by tractor-ploughing and ridging with a height of 35 cm and row spacing of 1.2 m × 0.5 m. All other field management practices adhered to the high-yield and quality cultivation measures of Fujian Province, with fertilization rates set at 127.5 kg·ha^−1^ N, 99 kg·ha^−1^ P₂O₅, and 402 kg·ha^−1^ K₂O. The application rates of the soil amendments were determined based on the results (Zhang B. et al., 2023; Zhang B. H. et al., 2023; Huang J. W. et al., 2023; Huang X. Q. et al., 2023).

During the tobacco growing season in 2021, the accumulated temperature was 2445.59°C and the total rainfall was 459.11 mm. In 2022, the accumulated temperature was 2209.04°C and the total rainfall was 610.43 mm. The initial soil pH was 4.96, with a soil organic matter content of 27.65 g·kg^−1^, total nitrogen of 2.06 g·kg^−1^, total phosphorus of 0.88 g·kg^−1^, total potassium of 24.91 g·kg^−1^, alkali-hydrolyzable nitrogen of 96.37 mg·kg^−1^, available phosphorus of 51.10 mg·kg^−1^, and available potassium of 190.06 mg·kg^−1^.

The biochar used in the amendment experiment was produced from tobacco stalks by Sanli New Energy Co., Ltd. in Shangqiu, Henan Province, through high-temperature pyrolysis (approximately 450°C) under anaerobic conditions. Its physicochemical properties are as follows: pH of 9.66, fixed carbon content of 475.90 g·kg^−1^, total nitrogen of 15.00 g·kg^−1^, total phosphorus of 1.40 g·kg^−1^, and total potassium of 20.10 g·kg^−1^. The lime used primarily consists of calcium oxide and calcium carbonate, with a pH of 11.41.

### Survey of tobacco bacterial wilt disease

During the tobacco harvest period, investigate the number of diseased plants under various treatments, classify the severity of the disease according to “GB/T 23222–2008” (Supplementary Table S1), and calculate the incidence percentage and disease index Equations 1, 2 (Cai et al., 2021).


(1) $$\begin{array}{l} \mathrm{Incidence percentage}\;\left(\%\right)=(\mathrm{Number of diseased plants}\\ \;/\mathrm{Total number of plants surveyed})\times 100\% \end{array}$$

(2) $$\begin{array}{l} \mathrm{Disease index}=\sum (\mathrm{number of diseased plants}\;\mathrm{at}\;\mathrm{each level}\\ \;\times \mathrm{corresponding grade value)}/(\mathrm{highest value}\\ \;\times \mathrm{total number of plants investigated})\times 100. \end{array}$$

### Survey of tobacco economic traits

The tobacco yield was individually measured based on the plot area during the harvesting period. The cured tobacco leaves were classified according to GB 2635–1992 “Flue-cured Tobacco,” and subsequently, the tobacco yield and output value of each plot were calculated.

### Soil physicochemical property determination

During the vigorous growth stages of tobacco, a strategically placed sampling point was positioned on the ridge surface between two representative tobacco plants in each plot. According to the five-point sampling method, five soil samples were collected from each plot. Subsequently, a single sample was provided for each repeating plot before mixing the soil of the same layer. Soil bulk density, porosity, and field capacity were subsequently measured at depths of 0–10 cm, 10–20 cm, and 20–30 cm in the topsoil using a ring knife method. The obtained measurements were averaged across three repetitions for each treatment (Zhang et al., 2023; Zhang B. H. et al., 2023). A total of 36 soil samples (3 × 4 × 3) are were collected from the 0–30 cm soil layer for determining soil nutrients. The samples were air-dried indoors and ground using a 1 mm mesh. pH is determined using the potentiometric method with an acidity meter. Soil exchangeable acidity is determined using the KCl exchange-neutralization titration method, and soil exchangeable bases are determined using the CH_3_COONH_4_ exchange-neutralization titration method. The sum of these two values represents the soil cation exchange capacity. The determination of total nitrogen, phosphorus, and potassium contents was conducted using the HClO_4_-H_2_SO_4_ digestion method with the assistance of the Smartchem 2000 fully automated discrete chemical analyzer and the NaOH fusion-flame photometry method. The soil’s organic matter content was measured through the H_2_SO_4_-K_2_Cr_2_O_7_ external heating method. Additionally, the levels of alkali-hydrolyzable nitrogen, available phosphorus, available potassium, and readily oxidisable organic carbon in soil were assessed using respective techniques such as alkali hydrolysis diffusion for nitrogen determination or molybdenum antimony anti-colorimetric for phosphorus measurement (Bao, 2000; Gu et al., 2023).

### Rhizosphere soil bacterial DNA extraction and high-throughput sequencing

During the vigorous growth period of tobacco, three representative tobacco plants exhibiting consistent growth patterns were chosen for each experimental treatment. The entire root systems of the tobacco plants were excavated, and the impurities surrounding the root system were eliminated. The soil samples adhered to the root surfaces were homogenized and wrapped in tin foil, followed by rapid freezing in liquid nitrogen and subsequent transfer to a –80°C freezer for storage. Soil DNA extraction, PCR amplification, and sequencing were all completed by Allwegene Technology Co., Ltd. (Beijing, China). After sampling soil and extracting total DNA of soil microorganisms, DNA samples were stored at –20°C following detection using NanoDrop 2000 UV–Vis spectrophotometer and agarose gel electrophoresis. Following total DNA extraction from soil, primers 338F (5’-ACTCCTACGGGAGGCAGCAG-3’) and 806R (5’-GGACTA CHVGGGTWTCTAAT-3’) with barcodes were used to amplify the V3-V4 region of 16S rDNA sequences. Library construction was performed using the TruSeq® DNA PCR-Free Sample Preparation Kit, and sequencing was carried out on the Illumina Miseq platform. Raw image data from sequencing were processed using the RDP Classifier algorithm and Silva database Release 119 to cluster reads with >97.0% similarity, generating operational taxonomic unit (OTU) representative sequences for comparative analysis. Species information of the community was annotated, and OTU abundance in each sample was normalized for diversity and differential analysis (NCBI accession number: PRJNA1118945).

### Bioinformatics analysis

After sequencing the 16S rDNA sequences, raw sequences were filtered and quality-checked using QIIME (v1.8.0). Operational taxonomic units (OTUs) were clustered at 97% similarity using Vsearch (v2.7.1). Clustering was performed with the Uparse method (Edgar, 2013), and denoising was carried out using the Unoise3 method (Rognes et al., 2016). To obtain species classification information for each OTU, the RDP Classifier algorithm was used to evaluate representative sequences with 70% confidence (Wang et al., 2007), and species information was annotated at various taxonomic levels (Kingdom, Phylum, Class, Order, Family, Genus, Species). After normalizing OTU abundance for each sample, diversity and differential analyses were conducted. Alpha species diversity was calculated using the diversity function from the R package “vegan” (Dixon, 2003), while phylogenetic diversity (PD), phylogenetic species variability (PSV), phylogenetic species richness (PSR), and phylogenetic species evenness (PSE) were computed using the R package “picante” (Kembel et al., 2010). Species diversity and phylogenetic diversity were standardized using Z-scores (Equation 3), where *x*_*i*_ represents the observed value for treatment *i*, and *μ* and *σ* denote the mean and standard deviation of the dataset.


(3) $$Z=\left(x_{i}-\mu \right)/\sigma$$

### Functional prediction of community species

The bacterial functions were categorized using the Plant-Beneficial Bacteria (PBB) database, which is constructed based on microbial taxonomy and functional traits (Li P. F. et al., 2023).

### Community ecology information and assembly mechanisms

The term “niche breadth” refers to the ecological position occupied by a population within an ecosystem and encompasses the entire spectrum of resources it utilizes. It is a key indicator for measuring the diversity of resource use and environmental adaptability of a species. A broader niche breadth indicates greater capability to exploit various resources and higher environmental adaptability, potentially leading to more stable survival across larger temporal or spatial scales. Niche breadth was calculated using the Levin method from the R package “spaa” (Zhang and Ma, 2013) (Equation 4), where *P_ij_* represents the proportion of the *i*-th species at sampling site *j*, and *r* is the total number of sampling sites. Microbial community stability was assessed using the Average Variation Degree (AVD) based on the method proposed by Xun et al. (2021) (Equation 5), where *k* is the number of samples in each group, *n* is the number of OTUs in each group, *a_i_* represents the variation of OTU*_i_*, and *x_i_* is the abundance of OTU*_i_* in a single sample after rarefaction. *x_i_* and *δ_i_* denote the mean and standard deviation of the abundances of OTU*_i_* across a group of samples after rarefaction. Typically, lower AVD values indicate greater community stability, while higher AVD values suggest increased community activity.


(4) $$\mathrm{Niche breadth}=1/r.\overset{r}{\underset{j=1}{\sum }}\left(P_{ij}\right)$$

(5) AVD=∑i=1n|ai|k×n,|ai|=xi−−xiδi

The C-score null model method proposed by Stone and Roberts (1990) was used to assess the randomness of species distributions. The observed C-score was calculated to evaluate species co-occurrence, and a null distribution of the simulated C-scores was generated using a randomization algorithm. By comparing the observed C-score with the null distribution, the standardized effect size (SES) was computed. Higher absolute values of SES indicate stronger relative contributions of deterministic processes. All calculations were performed using the “EcoSimR” package (Mo et al., 2021).

Null model analysis was conducted using the framework described by Stegen et al. (2012) to quantify the relative contributions of different treatments to microbial community assembly processes. Beta Nearest Taxon Index (βNTI) calculations were performed using the R package “picante” (Kembel et al., 2010). When βNTI < −2 or ≥ 2, it was classified as homogenizing or heterogeneous selection, respectively. For |βNTI| < 2, RCbray was calculated using the “vegan” package (Dixon, 2003). RCbray < −0.95 indicated homogenizing dispersal, RCbray ≥0.95 indicated dispersal limitation, and |RCbray| < 0.95 represented drift.

### Construction and characteristics of microbial community co-occurrence networks

The OTU table, after rarefaction, was used to independently construct microbial co-occurrence networks for each treatment. The microbial network matrices were created using the “reshape2” and “WGCNA” packages (Wickham, 2007; Langfelder et al., 2008), and network visualizations were generated in Gephi 0.10.1, retaining edges with Spearman correlation coefficients |*r*| > 0.65 and *p* < 0.01. To evaluate the complexity of microbial networks and interspecies interactions, we calculated various network features, including the number of nodes, number of edges, average degree, graph density, modularity index, and the proportion of positive to negative interactions, using the “igraph” package (Yuan et al., 2021). Cohesion is a measure of microbial interactions and was used to understand how positive/negative species interactions or niche similarities/differences affect community connectivity across temporal, spatial, or environmental gradients (Herren and McMahon, 2017). For a given network, the cohesion index is calculated as the sum of significant positive or negative correlations between species, weighted by species abundance (Equation 6), reflecting the extent of cooperative behavior or interactions, and is widely applied in various habitats.


(6) $$\mathrm{Cohesion}=\overset{n}{\underset{i=1}{\sum }}\mathrm{abundanc}e_{i}\times \mathrm{connectednes}s_{i}$$

### Statistical analysis

Principal Coordinates Analysis (PCoA) based on Bray-Curtis distance was performed using the corresponding functions from the “vegan” package (Dixon, 2003). The random forest regression model, built with the “randomForest” package, was employed to predict key soil properties influencing the incidence of tobacco bacterial wilt (Liaw and Wiener, 2002). Spearman’s rank correlation coefficient was used to analyze the relationships between community assembly processes, co-occurrence patterns, and tobacco bacterial wilt incidence. Graphs and charts were created using Microsoft Excel 2019, Origin 2024b, and R 4.2.9 software. Statistical significance was assessed using SPSS 22.0, with Duncan’s multiple range tests applied at *α* = 0.05.

## Results

### Effect of different soil amendments on the incidence of tobacco bacterial wilt disease and performance of agronomic traits

**Figure 1:**
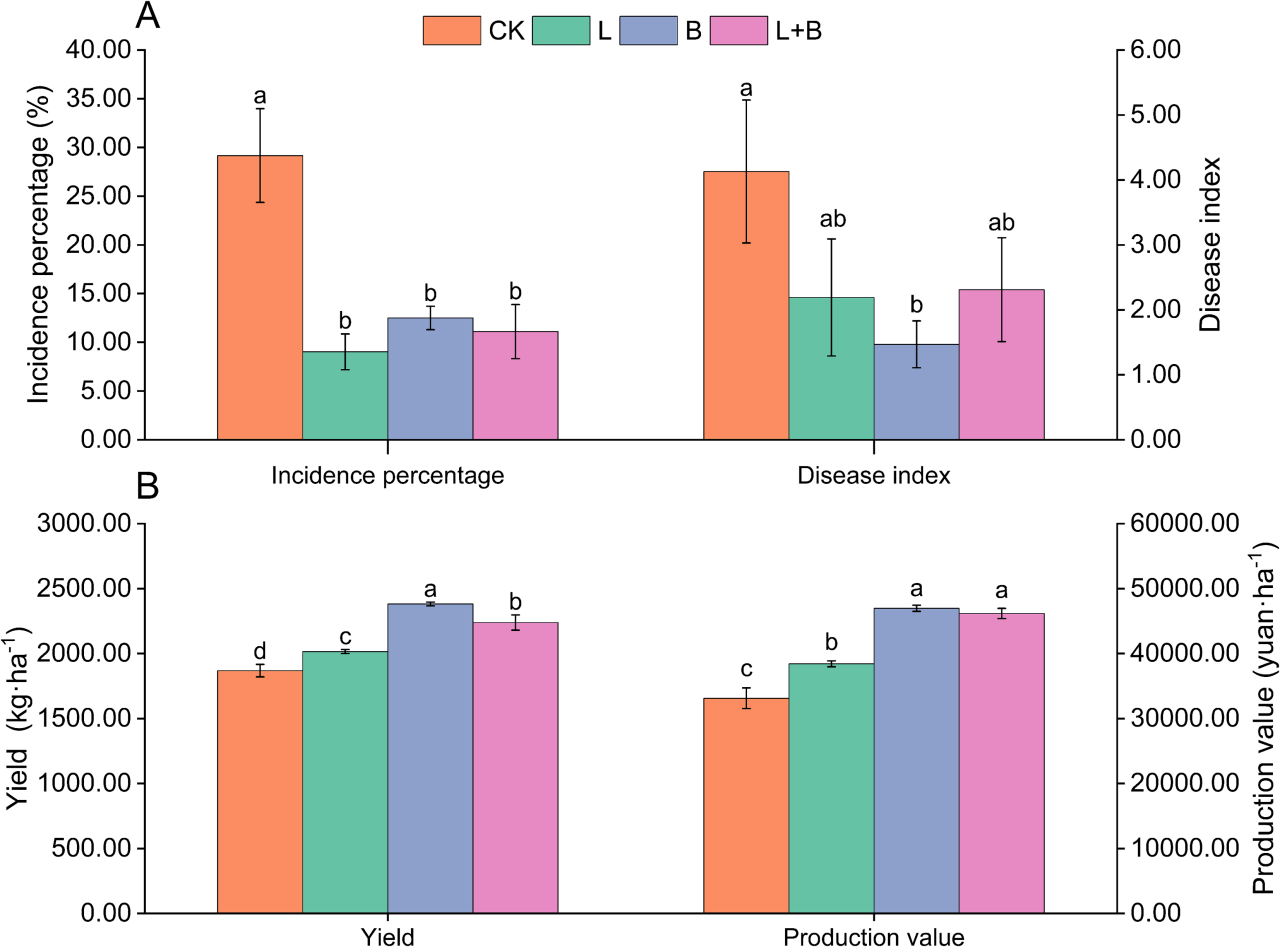
Incidence of bacterial wilt **(A)** and economic traits **(B)** of tobacco plants in each treatment (2022). CK, control without soil amendment; L, lime treatment; B, biochar treatment; L + B, lime and biochar mixture treatment. Data depicts means ± SD of three biological replicates. Significant differences between treatments (*p* < 0.05) are illustrated by different lowercase letters.

As shown in Supplementary Table S2, in the 2021 experiment, the application of lime or biochar (L, B) significantly reduced the incidence percentage and disease index of tobacco bacterial wilt disease compared to the CK treatment. However, in terms of yield and production value, the B treatment outperformed the L treatment. The results from 2022 (Figure 1) indicate that compared to the CK treatment, each treatment significantly reduced the incidence percentage of tobacco bacterial wilt disease by 16.67 to 20.14% (*p* < 0.05) and significantly increased tobacco yield by 7.86 to 27.46% (*p* < 0.05). However, only the B treatment significantly reduced the disease index compared to the CK treatment. Moreover, in terms of yield and production value, the B treatment showed the best improvement, followed by the lime and biochar mixture treatment (L + B). The findings from the 2-year experiment suggested that various soil amendments exhibit significant disease suppression effects; however, they demonstrate diverse impacts on tobacco yield and its economic value.

### Changes in the physicochemical properties of tobacco-growing soil and their correlation with the incidence of bacterial wilt disease

As shown in Table 1, compared to the CK treatment, the application of soil amendments significantly increased soil pH, reduced exchangeable acidity, and significantly increased exchangeable base content and cation exchange capacity (*p* < 0.05). In terms of soil bulk density and field capacity, both B and L + B treatments significantly decreased soil bulk density and increased field capacity compared to the CK treatment (*p* < 0.05), while only the lime treatment (L) showed no significant difference in bulk density and field capacity compared to the CK treatment. Regarding soil nutrients, both B and L + B treatments significantly increased soil alkali-hydrolyzable nitrogen, available phosphorus, available potassium, and readily oxidisable organic content compared to the CK treatment (*p* < 0.05), while the L treatment only significantly increased available potassium content (*p* < 0.05). The results suggest that the application of lime alone exhibits limited efficacy in soil improvement, whereas the combination of lime with biochar demonstrates a multifaceted enhancement in soil physicochemical properties.

**Table 1:** Physicochemical properties of tobacco soil in each treatment.

	CK	L	B	L + B
Bulk density (g·cm^−3^)	1.37 ± 0.03a	1.38 ± 0.04a	1.16 ± 0.01c	1.24 ± 0.01b
Porosity (%)	48.44 ± 0.12c	47.94 ± 0.16c	56.14 ± 0.24a	53.38 ± 0.41b
Field capacity (%)	31.75 ± 0.29c	31.49 ± 0.27c	43.95 ± 0.28a	41.78 ± 0.35b
pH	4.89 ± 0.01d	5.04 ± 0.02c	5.27 ± 0.01a	5.13 ± 0.02b
Exchangeable acidity (mmol·kg^−1^)	12.83 ± 0.17a	8.33 ± 0.01b	7.00 ± 0.17c	7.17 ± 0.17c
Exchangeable bases (mmol·kg^−1^)	29.84 ± 0.04c	35.50 ± 0.07b	37.12 ± 0.11a	37.34 ± 0.04a
Cation exchange capacity (mmol·kg^−1^)	42.67 ± 0.15c	43.84 ± 0.11b	44.32 ± 0.11a	44.50 ± 0.19a
Total nitrogen (g·kg^−1^)	2.15 ± 0.70a	2.21 ± 0.33a	2.18 ± 0.20a	2.19 ± 0.26a
Alkali-hydrolyzable nitrogen (mg·kg^−1^)	169.67 ± 3.33b	170.00 ± 5.04b	190.33 ± 2.33a	186.83 ± 5.41a
Total phosphorus (g·kg^−1^)	0.90 ± 0.02a	0.92 ± 0.02a	0.91 ± 0.04a	0.92 ± 0.02a
Available phosphorus (mg·kg^−1^)	68.58 ± 1.77b	71.27 ± 1.32b	79.72 ± 2.58a	75.56 ± 1.08a
Total potassium (g·kg^−1^)	25.84 ± 0.10c	26.39 ± 0.49bc	27.48 ± 0.11a	26.82 ± 0.30b
Available potassium (mg·kg^−1^)	314.72 ± 7.16d	334.44 ± 5.68c	387.98 ± 7.06a	355.06 ± 7.52b
Soil organic carbon (g·kg^−1^)	16.30 ± 0.10b	16.49 ± 0.19b	16.94 ± 0.20a	16.75 ± 0.10a
Readily oxidisable organic carbon (g·kg^−1^)	3.14 ± 0.14c	3.05 ± 0.07c	4.20 ± 0.16a	3.86 ± 0.06b

CK, control without soil amendment; L, lime treatment; B, biochar treatment; L + B, lime and biochar mixture treatment. Data depicts means ± SD of three biological replicates. Significant differences between different treatments (*P* < 0.05) are illustrated by different lowercase letters.

**Figure 2:**
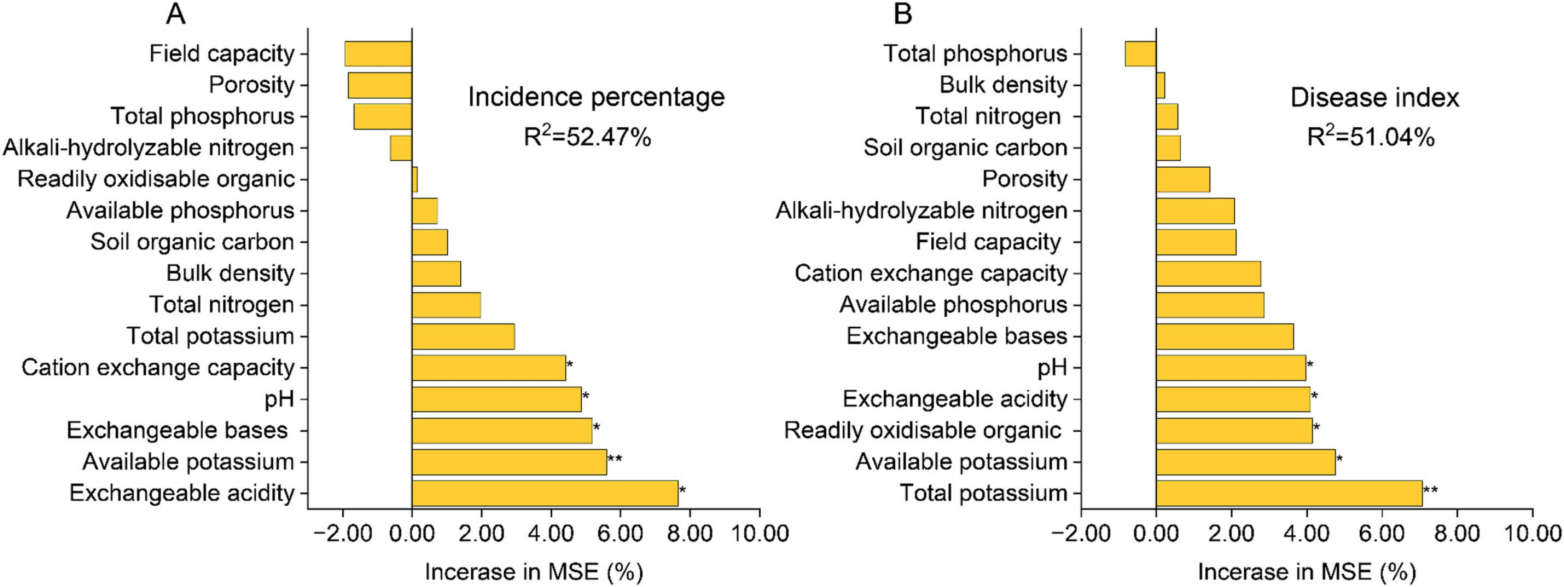
Random Forest regression model predicts the correlation between soil physicochemical properties and the incidence percentage of tobacco bacterial wilt **(A)** as well as the disease index **(B)**. The precision importance measures were calculated for each tree in a random forest and averaged over the entire forest (5,000 trees). The percentage increase in the mean squared error (MSE) of the variables was used to estimate the importance of these predictors. “*”at 0.05 level (two-tailed), the correlation was significant; “**” at level 0.01 (two-tailed), the correlation was significant. CK, control without soil amendment; L, lime treatment; B, biochar treatment; L + B, lime and biochar mixture treatment.

The random forest regression model, as depicted in Figure 2, demonstrates that soil physicochemical properties exhibit a strong predictive capacity for the incidence percentage and disease index of tobacco bacterial wilt disease, with a total explanation rate exceeding 50%. Available potassium, pH level, and exchangeable acidity display significant correlations with both the incidence percentage and disease index (*p* < 0.05). Cation exchange capacity and exchangeable bases are significantly associated with the disease incidence percentage (*p* < 0.05), while readily oxidizable organic matter exhibits a significant correlation with the disease index (*p* < 0.05).

### Changes in rhizosphere bacterial community diversity

To investigate the dynamic characteristics of rhizosphere microbiota in response to reduced incidence of bacterial wilt disease, high-throughput sequencing of the V3-V4 region was performed using the Illumina MiSeq platform. A total of 1,867,236 bacterial 16S rDNA sequences were identified, resulting in 9201 operational taxonomic units (OTUs). The rarefaction curves of OTUs at a 97% sequence similarity level exhibited saturation without further increase (Supplementary Figure S1A), indicating that the data accurately represented the composition of rhizosphere bacterial communities and facilitated subsequent data analysis.

The effect of soil amendments on bacterial community species diversity and phylogenetic diversity (Supplementary Figure S1B). Both L and L + B treatments significantly increased species diversity (Observed OTUs, Ace, Chao1, Shannon) and phylogenetic diversity (PD, PSR, PSE) compared to the CK treatment (*p* < 0.05), whereas the B treatment showed no significant difference from the CK treatment in species diversity and phylogenetic diversity.

As depicted in Supplementary Figure S1C, PCoA based on Bray-Curtis distance quantified differences in species composition among treatments, with a cumulative explanatory rate of 63.71%. Only the B treatment significantly separated from the CK treatment along PCoA1 and PCoA2 axes (PERMANOVA, *R*^2^ = 61.57%, *p* = 0.001). However, L and L + B treatments exhibited significant separation from the CK treatment only along the PCoA2 axis (PERMANOVA, *R*^2^ = 61.57%, *p* = 0.001). It is evident that different soil amendments have inconsistent effects on species diversity and composition of rhizosphere bacterial communities.

### Changes in the species composition of bacterial communities and the abundance of *Ralstonia* in rhizosphere soil

**Figure 3:**
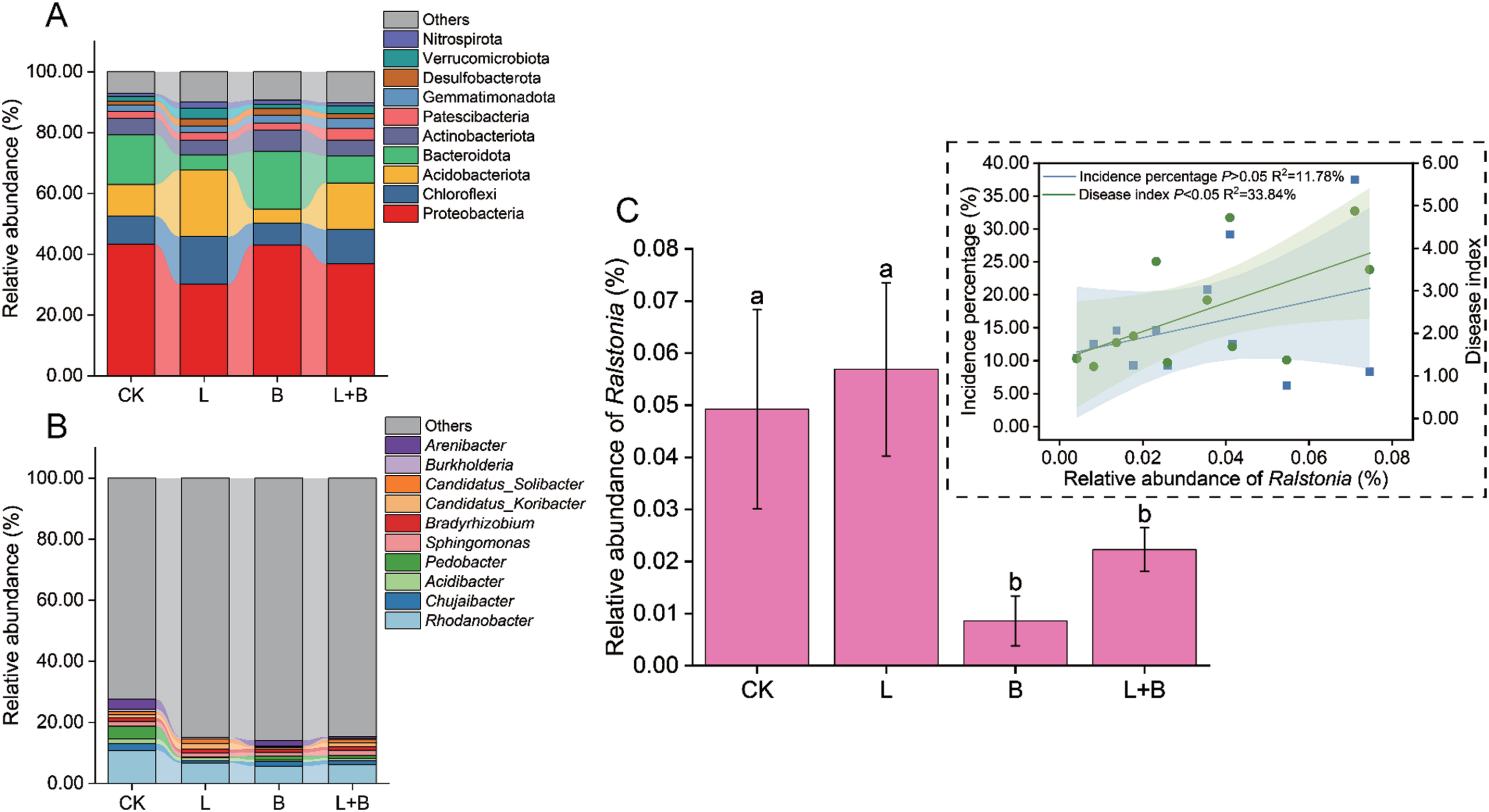
Alterations in the relative abundance of dominant bacterial phyla **(A)** and genera **(B)** in the rhizosphere of tobacco, changes in the relative abundance of *Ralstonia*, and its Pearson correlation analysis with tobacco bacterial wilt incidence **(C)**. Both dominant phyla and genera were selected based on the top 10 in relative abundance. CK, control without soil amendment; L, lime treatment; B, biochar treatment; L + B, lime and biochar mixture treatment. Data depicts means ± SD of three biological replicates. Significant differences between treatments (*p* < 0.05) are illustrated by different lowercase letters.

In this sequencing, a total of 59 bacterial phyla and 818 bacterial genera were annotated. The top 10 dominant bacterial phyla or genera in relative abundance were selected for comparison. The results indicated that different soil amendments had inconsistent effects on the relative abundance of dominant bacterial phyla in rhizosphere bacterial communities (Figure 3A). The L and L + B treatments significantly reduced the relative abundance of Proteobacteria and Bacteroidota, while significantly increasing the relative abundance of Chloroflexi and Acidobacteriota (*p* < 0.05). Conversely, the B treatment significantly increased the relative abundance of Proteobacteria and Bacteroidota, while significantly decreasing the relative abundance of Chloroflexi and Acidobacteriota (*p* < 0.05). With respect to the dominant bacterial genera (Figure 3B), a significant reduction in the relative abundance of *Rhodanobacter* and *Chujaibacter* was detected following the application of soil amendments.

As shown in Figure 3C, compared to the CK treatment, the B and L + B treatments significantly reduced the relative abundance of the main pathogen of bacterial wilt, *Ralstonia*, but there was no significant change compared to the L treatment and CK treatment. Pearson linear regression analysis indicated that the relative abundance of *Ralstonia* was not significantly correlated with the incidence percentage of bacterial wilt (*p* > 0.05) but was significantly positively correlated with the disease index (*p* < 0.05). However, its explanatory power for the disease severity index was only 33.84% (R^2^ = 33.84%).

### Functional prediction of rhizosphere bacterial communities and their assembly mechanisms

Based on the PBB database, the prediction of the tobacco rhizosphere bacterial community was predicted (Supplementary Figure S2). The results indicated that compared to the CK treatment, the L treatment significantly decreased the relative abundance of PGPR (Plant Growth-Promoting Rhizobacteria), Plant pathogen bacteria, Biocontrol bacteria, and Stress resistance bacteria (*p* < 0.05). However, there was no significant change in these parameters compared to the CK treatment for the B treatment and L + B treatment.

**Figure 4:**
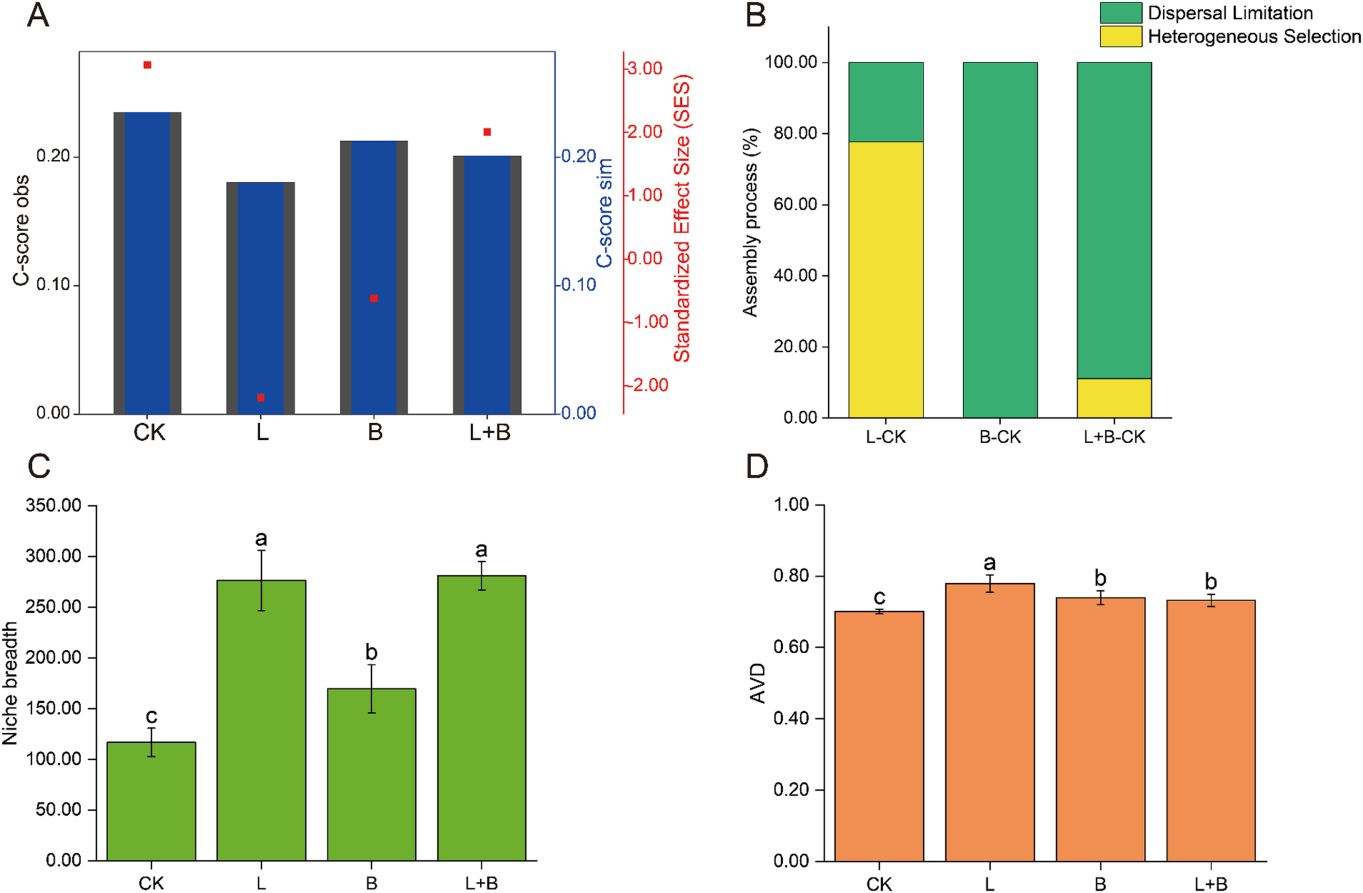
Mechanisms of rhizosphere bacterial community assembly, calculated using a phylogenetic null model C-score **(A)** and the relative contributions of different assembly processes to the community assembly mechanisms **(B)**, community niche breadth **(C)**, and AVD **(D)**. C-score metric using null models. The values of observed C-score (C-score_obs_) > simulated C-score (C-score_sim_) indicate non-random co-occurrence patterns. Standardized effect size <−2 and >2 represent aggregation and segregation, respectively. AVD, average variation degree; CK, control without soil amendment; L, lime treatment; B, biochar treatment; L + B, lime and biochar mixture treatment. Data depicts means ± SD of three biological replicates. Significant differences between treatments (*p* < 0.05) are illustrated by different lowercase letters.

The community assembly mechanisms, as shown in Figure 4, were illustrated through the C-score results. A higher |SES| value indicates a stronger relative contribution of deterministic processes, with the magnitude of |SES| ranked as CK > L > L + B > B (Figure 4A). Using the Beta Nearest Taxon Index (βNTI), the relative contributions of deterministic and stochastic community assembly processes to changes in rhizosphere bacterial communities after applying soil amendments were quantified (Figure 4B). The results indicated that compared to the CK treatment, the assembly process of rhizosphere bacterial communities in the L treatment was primarily dominated by heterogeneous selection (77.78%), while the B treatment and L + B treatment were dominated by dispersal limitation (100, 88.89%). Regarding the ecological niche breadth and average variation degree (AVD) (Figures 4C,D), consistent results were observed across different soil amendment treatments, all showing significant improvements compared to the CK treatment. The ecological niche breadth increased significantly by 45.16 to 140.71% (*p* < 0.05), and AVD increased significantly by 4.45–11.15% (*p* < 0.05).

### Co-occurrence patterns and the co-evolutionary rules of rhizosphere bacterial communities

**Figure 5:**
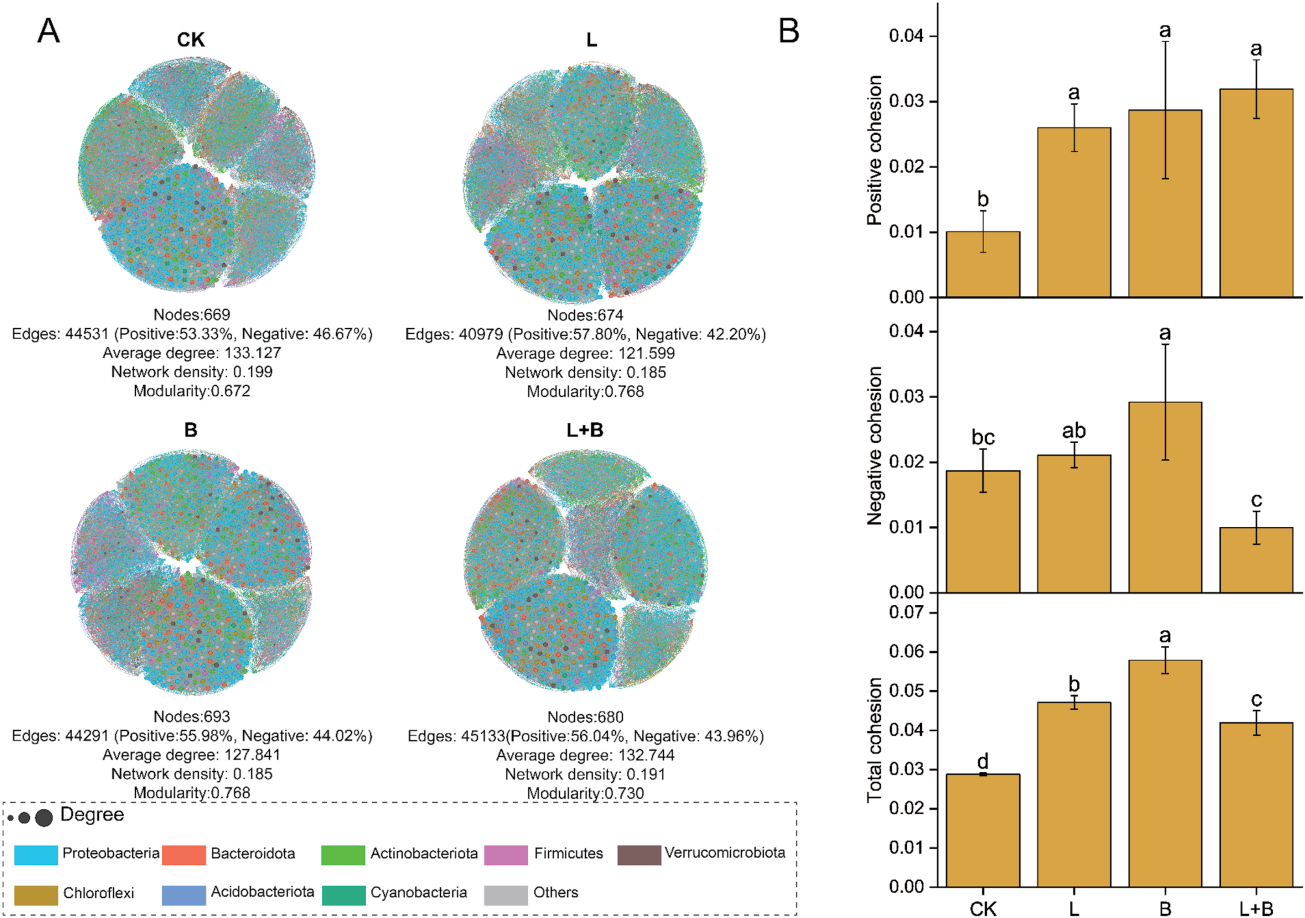
Co-occurrence network analysis **(A)** and community cohesion **(B)** of rhizosphere bacterial communities under each treatments. Each node represents a distinct bacterial genus, with node size corresponding to its degree, nodes are colored based on their phylum in the co-occurrence network. CK, control without soil amendment; L, lime treatment; B, biochar treatment; L + B, lime and biochar mixture treatment. Data depicts means ± SD of three biological replicates. Significant differences between treatments (*p* < 0.05) are illustrated by different lowercase letters.

By constructing a bacterial community network to understand the co-evolutionary rules within communities, the results are shown in Figure 5A, indicating that each treatment has increased the modularity index and reduced the graph density in the community network compared to the CK treatment, while significantly increasing the proportion of positive interactions among species. Based on species interactions and species abundance-weighted calculations of community cohesion, the results are shown in Figure 5B, indicating that each treatment significantly enhanced both positive and total community cohesion compared to the CK treatment (*p* < 0.05).

**Figure 6:**
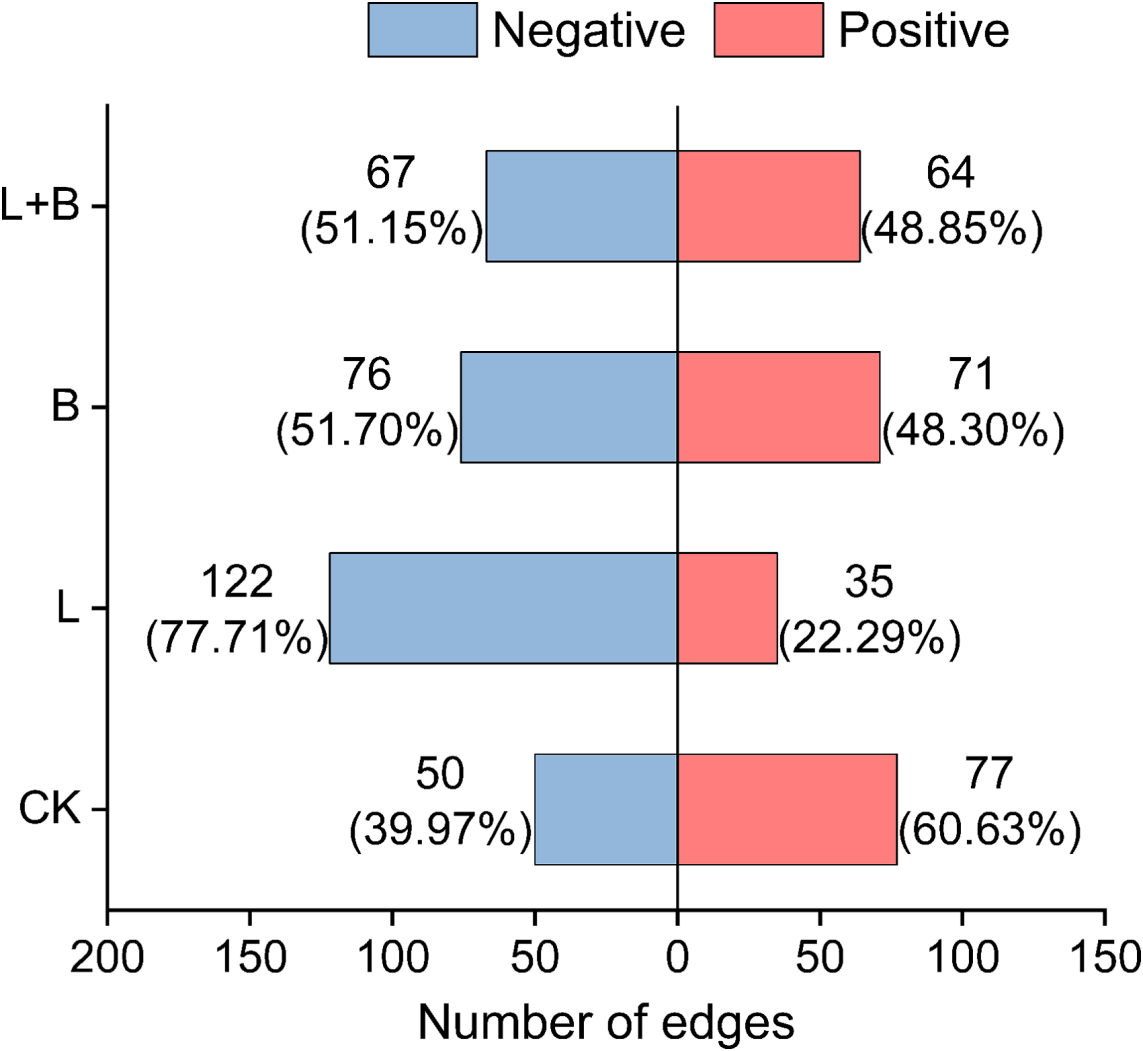
In the co-occurrence network analysis of rhizosphere bacterial communities, the interactions between Ralstonia and other bacterial genera are investigated. CK, control without soil amendment; L, lime treatment; B, biochar treatment; L + B, lime and biochar mixture treatment.

Further analysis of the interactions of *Ralstonia* within the community, as shown in Figure 6, reveals that under the CK treatment, *Ralstonia* interactions within the community are mainly positive (60.63%), with negative interactions accounting for 39.97%. However, after the application of soil amendments, *Ralstonia* interactions within the community are predominantly negative (77.71, 51.70, 51.15%).

**Figure 7:**
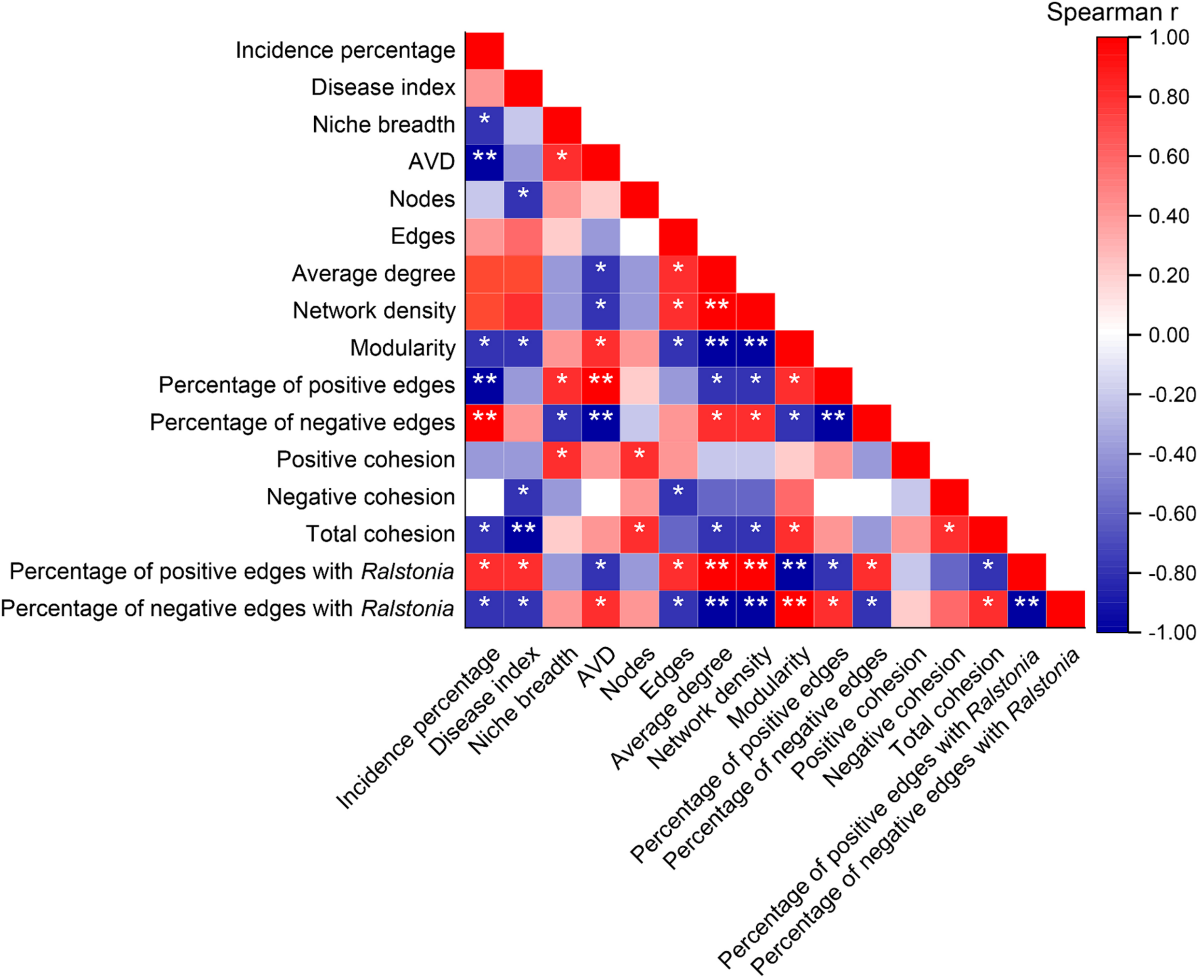
Correlations between mechanisms of community assembly and co-occurrence patterns with the incidence of bacterial wilt disease. “*” at 0.05 level (two-tailed), the correlation was significant; “**” at level 0.01 (two-tailed), the correlation was significant. AVD: average variation degree. CK, control without soil amendment; L, lime treatment; B, biochar treatment; L + B, lime and biochar mixture treatment.

Spearman correlation analysis indicated (Figure 7) that the incidence percentage of tobacco bacterial wilt is significantly negatively correlated with niche breadth, network modularity index, and total cohesion (*p* < 0.05). Additionally, the total cohesion is highly negatively correlated with the disease index (*p* < 0.01). There is a highly significant negative correlation between the incidence rate and community average variation degree (AVD) as well as the proportion of positive interactions within the network (*p* < 0.01), whereas it is highly positively correlated with the proportion of negative interactions (*p* < 0.01). Moreover, the proportion of positive interactions of *Ralstonia* within the community shows a significant positive correlation with both the incidence percentage and disease index (*p* < 0.05), while its proportion of negative interactions is significantly negatively correlated with these metrics (*p* < 0.05).

## Discussion

Soil acidification is a critical environmental factor influencing the occurrence of bacterial wilt disease, with outbreaks being particularly severe in tobacco-growing regions of southern China where acidification issues are increasingly pronounced (Li et al., 2017). Previous studies have demonstrated that the application of lime or biochar can effectively reduce the incidence of tobacco bacterial wilt disease (Shen et al., 2018; Li et al., 2022). Our two-year field experiment revealed that the application of lime (L), biochar (B), or their combination (L + B) significantly reduces the incidence of tobacco bacterial wilt (*p* < 0.05) (Supplementary Table S2 and Figure 1A). However, in terms of disease severity and economic traits, the B treatment performed better than both the L and L + B treatments (Figures 1A,B). This may be attributed to biochar’s additional benefits over lime; besides alleviating soil acidification like lime, biochar’s high carbon content, good porosity, strong adsorption capacity, and rich nutrient profile provide a superior “refuge” for microbial habitation and proliferation. It supplies diverse carbon sources, energy, and mineral nutrients, which enhances microbial growth, consequently, modifies the microenvironment of crop growth. This improved microenvironment increases the efficiency of nutrient utilization and disease resistance in crops (Zhang et al., 2021). These findings align with the results of Xie et al. (2013) and Azim et al. (2024), underscoring the importance of selecting appropriate materials for soil remediation and plant disease management.

The exacerbation of soil acidification is often accompanied by the loss of base cations and nutrients in the soil, as well as the accumulation of H^+^ and Al^3+^ (Dong et al., 2022). This evidently threatens the plant disease defense mechanisms. The results of this study indicate that the biochar (B) and lime plus biochar (L + B) treatments significantly increased the levels of effective nutrients such as alkali-hydrolyzable nitrogen and readily oxidisable organic carbon, and decreased soil bulk density compared to the control (CK) (*p* < 0.05), while the lime (L) treatment did not show significant differences from the CK treatment (Table 1). All treatments significantly improved soil pH, exchangeable acidity, exchangeable bases, and available potassium compared to the CK treatment (*p* < 0.05) (Table 1), which is consistent with findings by Zhang C. et al. (2017) and Liang et al. (2021). As a major abiotic factor affecting plant disease incidence, increased soil pH can directly or indirectly impact the survival environment of plant pathogens. For instance, higher pH directly inhibits the metabolic activity of *Ralstonia solanacearum* and reduces its colonization rate in the soil (Huang J. W. et al., 2023; Huang X. Q. et al., 2023). Additionally, alkaline soil conditions promote the growth of actinomycetes and certain antagonistic fungi, which produce natural antibiotics or other metabolites that help resist pathogen invasion (Bibi et al., 2018; Lee et al., 2021). Moreover, exchangeable base cations in the soil directly benefit plant root health by aiding in more effective water and nutrient absorption, enhancing cellular structure, and improving disease resistance (Sharma et al., 2018; Niu et al., 2021). Li et al. (2017) demonstrated that increased soil pH enhances the expression of resistance genes in the soil, playing a crucial role in suppressing bacterial wilt. Predictions from the random forest regression model in this study indicate that soil pH, exchangeable acidity, exchangeable bases, and available potassium are the primary soil physicochemical factors affecting the incidence and severity of tobacco diseases (*p* < 0.05) (Figures 2A,B). Thus, increasing soil pH and concentrations of exchangeable bases (Ca^2+^, K^+^, Na^+^, Mg^2+^) while reducing exchangeable acidity (Al^3+^, H^+^) are core strategies for managing bacterial wilt, and maintaining appropriate levels of available nutrients in the soil is essential to ensure crop yield.

The composition of plant rhizosphere microorganisms is an inheritable trait and represents secondary genomics that exert influences on plant growth, development, as well as yield and quality. Soil-borne diseases are closely related to the diversity and species composition of rhizosphere microbial communities (Ling et al., 2022). Several studies have indicated that soil acidification primarily reduces soil suppressive capacity against pathogens by regulating bacterial community structure (Chen et al., 2022; Li X. et al., 2023). Previous studies have demonstrated that the application of lime or biochar to enhance soil pH leads to an increase in soil microbial community diversity and induces alterations in microbial community composition (Gul et al., 2015; Guo et al., 2019). In this study, compared to the control (CK) treatment, the lime (L) and lime mixed biochar (L + B) treatments significantly increased bacterial community species diversity and phylogenetic diversity (Supplementary Figure S1B) (*p* < 0.05). The differences in rhizosphere soil bacterial composition between these treatments and the CK treatment, however, were not as pronounced as those observed with the biochar (B) treatment (Supplementary Figure S1C) (PERMANOVA, *R*^2^ = 61.57%, *p* = 0.001). Notably, there were no significant differences between the B treatment and the CK treatment in terms of community species diversity and phylogenetic diversity. Previous studies have suggested a significant positive correlation between disease severity in plant hosts and pathogen abundance (Zhang H. C. et al., 2017; Qi et al., 2022). In this study, the effects of different treatments on the relative abundance of *Ralstonia* were inconsistent; the L treatment did not significantly reduce the relative abundance of *Ralstonia* compared to the CK treatment (*p* < 0.05) (Figure 3C). Additionally, Pearson linear regression analysis revealed no significant correlation between the relative abundance of *Ralstonia* and bacterial wilt incidence (*p* < 0.05) (Figure 3C). Furthermore, functional classification of bacterial communities using the PBB database showed no consistent impact on the abundance of bacterial functional groups compared to the CK treatment, with both the B and L + B treatments not significantly reducing the relative abundance of plant pathogens compared to the CK treatment (Supplementary Figure S2). This result aligns with Yuan et al. (2020), who argued that relying solely on the relative abundance of pathogens, such as *Fusarium*, is insufficient for predicting the likelihood of plant disease. Based on these findings, we hypothesize that the key to suppressing bacterial wilt may involve not only reducing the abundance of *Ralstonia* within bacterial communities but also altering the abundance of various functional groups. This finding that our understanding of the rhizosphere microbial mechanisms in suppressing tobacco bacterial wilt still requires further investigation.

In natural ecosystems, the interactions among microbial species form highly dynamic and complex co-occurrence patterns (Hu et al., 2022). The evolution of community ecology research has rendered simplistic community models inadequate in capturing the entirety of community dynamics. A deeper understanding of community assembly mechanisms is crucial for exploring how different ecological processes shape and maintain microbial community composition and diversity patterns in varying soil environments (Stegen et al., 2012). In this study, treatments with soil amendments significantly reduced the relative contribution of deterministic processes to community assembly compared to the control (Figure 4A), indicating a decrease in environmental constraints on bacterial communities (Chase and Myers, 2011). βNTI analysis revealed that the assembly processes of bacterial communities differed by treatment: lime application (L) was primarily driven by heterogeneous selection, whereas biochar application (B) was mainly influenced by dispersal limitation (Figure 4B). Lime affects soil microbial community structure primarily by altering the microbial habitat, while biochar impacts community structure by increasing species turnover rates (Moeller et al., 2017), which is beneficial for improving community structure. Niche breadth measures a species’ functional role and status within the community, and a wider niche breadth generally indicates faster species migration and greater environmental adaptability (Slatyer et al., 2013). Community average variation degree (AVD) reflects the activity level of the community (Xun et al., 2021). During plant host infection, soil pathogens must compete for niches or resources with other microorganisms in the rhizosphere (Liu et al., 2021). In this study, all treatments significantly increased community niche breadth and AVD compared to the control (*p* < 0.05) (Figures 4C,D). Spearman correlation analysis showed a significant negative correlation between community niche breadth and disease incidence (*p* < 0.05) (Figure 7), and an extremely significant negative correlation between community AVD and disease incidence (*p* < 0.01) (Figure 7). Thus, we hypothesize that during the suppression of bacterial wilt, the significant increase in soil pH reduces environmental selective pressures on bacteria, improves the rhizosphere nutrient environment, enhances species migration rates, and decreases interspecies competition.

Microbial ecological interactions are crucial for maintaining the stability and functionality of soil microbial communities (Yang et al., 2017). Various soil microbial groups can interact through mutualistic, synergistic, commensal, or parasitic relationships, forming a complex and interconnected ecological network that directly impacts soil and plant health (Van Der Heijden and Hartmann, 2016). Research indicates that a higher diversity of microbial species within a network increases the likelihood of metabolic and informational exchanges among microbes (Qi et al., 2019). Gu et al. (2017) demonstrated that beneficial interspecies relationships are primarily characterized by positive correlations, which promote stable coexistence or mutualistic interactions. This study constructed a community interaction network to reveal the connections and interactions between previously independent microbial communities in the soil (Rillig et al., 2016). The results showed that all treatments significantly increased the number of nodes and the modularity index in the community network compared to the control, as well as the proportion of positive interactions among species (Figure 5A). Qiao et al. (2024) combined network and cohesion analyses and found that microbial networks in healthy soils exhibit higher stability and complexity, which in turn reduce plant disease indices and increase biomass. Additionally, cohesion, which reflects the level of cooperation, also showed a negative correlation with plant disease indices. Our study also found that all treatments significantly enhanced community positive cohesion and total cohesion compared to the control (*p* < 0.05) (Figure 5B). Correlation analysis further revealed significant negative correlations between network modularity index and total cohesion with disease incidence and disease index (*p* < 0.05) (Figure 7). After increasing soil pH, bacterial communities underwent niche differentiation, disrupting and reshaping existing interspecies relationships, thereby enhancing mutualistic interactions. This could be a key rhizomicrobial mechanism in suppressing tobacco bacterial wilt. The previous results showed no significant correlation between the relative abundance of *Ralstonia* and disease incidence (Figure 3C). To further elucidate *Ralstonia*’s role in community co-evolution during disease suppression, we examined co-occurrence relationships between *Ralstonia* and other species in the network. Under the control treatment, *Ralstonia* established more mutualistic positive interactions, while after lime or biochar application, its interactions shifted to competitive or antagonistic relationships (Figure 6). Zhang et al. (2022) suggested that alleviating bacterial wilt can be achieved by recruiting species that antagonize the pathogen, which is supported by our correlation analysis results (Figure 7). Therefore, the key to suppressing tobacco bacterial wilt lies in enhancing interspecies cooperation within the community, strengthening antagonistic relationships with pathogens, and promoting positive evolutionary changes in rhizomicrobial communities. The incorporation of this approach is pivotal in simultaneously enhancing plant productivity and soil fertility. Consequently, the development of efficient control technologies for tobacco bacterial wilt should thoroughly consider these ecological mechanisms to attain optimal integrated prevention outcomes.

## Conclusion

In summary, all amendment treatments significantly reduced the incidence of tobacco bacterial wilt and improved tobacco economic traits, with biochar (B) treatment being the most effective and lime + biochar (L + B) treatment being the second most effective. Improving soil pH and exchangeable base concentrations while reducing exchangeable acidity is a core strategy for managing bacterial wilt. Additionally, this study found that the lime + biochar (L + B) treatment more effectively improved soil physicochemical properties compared to lime alone (L). Therefore, this combined approach is recommended for balancing both ecological and economic benefits. Furthermore, in the context of bacterial wilt control mechanisms, the key is not only reducing *Ralstonia* abundance and altering the abundance of different functional groups but also enhancing the rhizosphere nutrient environment. This includes broadening the bacterial community’s ecological niche, reducing environmental pressures on bacterial communities, and fostering mutually beneficial symbiotic relationships while recruiting antagonistic species against *Ralstonia* to establish a stable rhizosphere microbial diversity mechanism. The results suggest that further exploration of *Ralstonia* antagonistic mechanisms in soil is needed, with targeted selection of beneficial antagonistic microbial communities suitable for various soil environments. Additionally, employing soil ecological enhancement agents containing diverse antagonistic microorganisms can accelerate the positive evolution of soil microbial communities and restore the diversity and health of soil microbiomes. These findings offer new research perspectives for developing ecological control strategies for *Solanaceous* crop bacterial wilt.

## Data Availability

The datasets presented in this study can be found in online repositories. The names of the repository/repositories and accession number(s) can be found in the article/Supplementary material.
