# Supplementary material for: Synergistic co-evolution of rhizosphere bacteria in response to acidification amelioration strategies: impacts on the alleviation of tobacco wilt and underlying mechanisms
Source: Front Microbiol. 2024 Oct 1;15:1448950. doi: 10.3389/fmicb.2024.1448950 (PMC11473436; doi:10.3389/fmicb.2024.1448950)
Supplement: Supplementary file 1 [file Data_Sheet_1.pdf]

Table S1 Disease rating scale to record disease index

Rating scale	Symptoms
0	No symptom
1	Below one half of tobacco leaves wilted and necrotic spots produced up to 1-20% of stem
3	One half to two-thirds of tobacco leaves wilted and necrotic spots reached up to 40% of stem
5	Above two-thirds of tobacco leaves wilted and necrotic spots reached up to 60% of stem
7	All leaves wilted and necrotic spots reached up to 80% of stem
9	Stems collapsed or tobacco plant died and necrotic spots reached up to 100% of stem

Table S2 Incidence of bacterial wilt and economic traits of tobacco plants in each treatment (2021)

	Incidence percentage (%)	Disease index	Yield ($kg\cdot ha^{-1}$)	Production value ($yuan\cdot ha^{-1}$)
CK	37.42±1.90a	14.14±1.03a	2013.77±27.47c	37922.25±535.52c
L	7.77±1.52b	2.75±0.59b	2438.75±18.01b	46702.06±847.58b
B	8.48±0.91b	3.08±0.73b	2867.13±95.04a	57100.22±1847.68a

CK: control without soil amendment; L: lime treatment; B: biochar treatment. Data depicts means ± SD of three biological replicates. Significant differences between treatments ($P < 0.05$) are illustrated by different lowercase letters

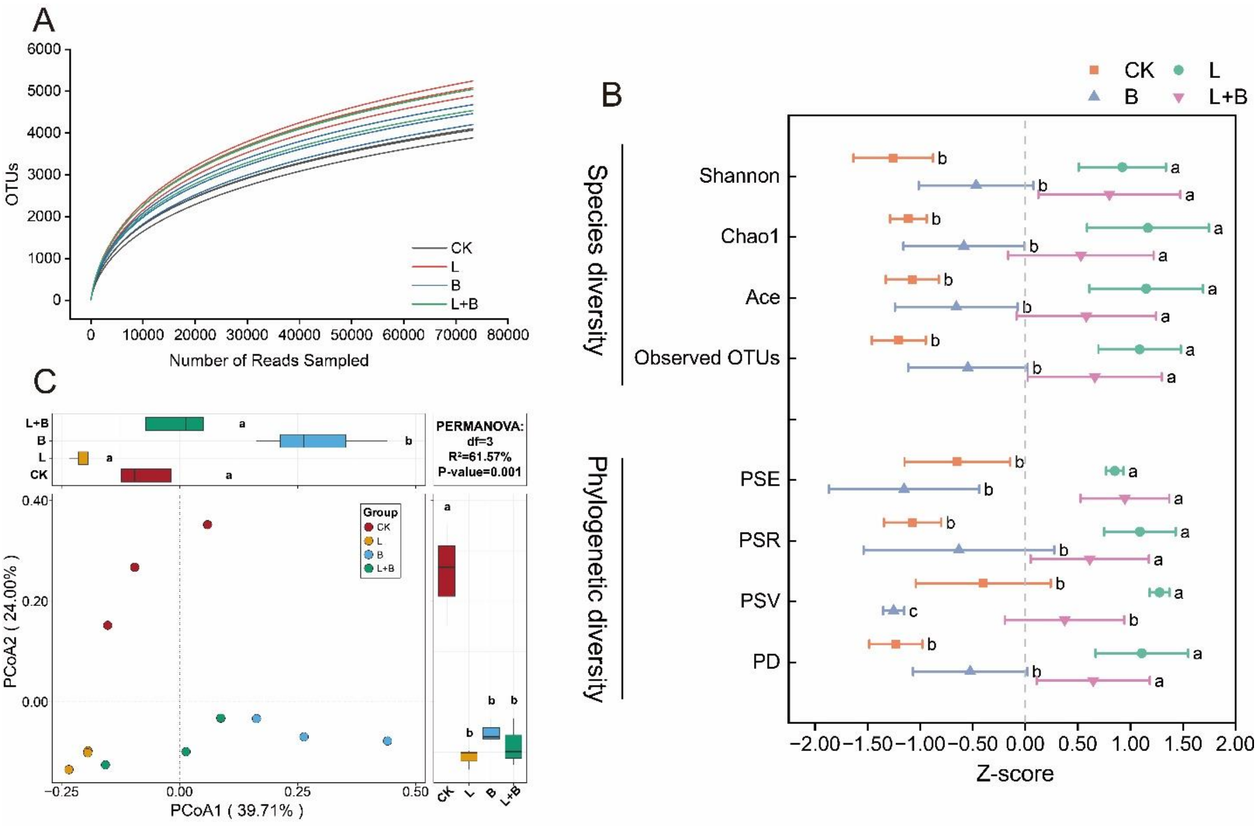

Fig.S1 Tobacco rhizosphere soil bacterial community OTUs rarefaction curve (A), community species diversity and phylogenetic diversity (B), community species composition PCoA analysis based on Bray-Curtis distance (C). PD: Phylogenetic diversity; PSV: Phylogenetic species variability; PSR: Phylogenetic species richness; PSE: Phylogenetic species evenness. CK: control without soil amendment; L: lime treatment; B: biochar treatment; L+B: lime and biochar mixture treatment. Data depicts means ± SD of three biological replicates. Significant differences between treatments ($P < 0.05$) are illustrated by different lowercase letters

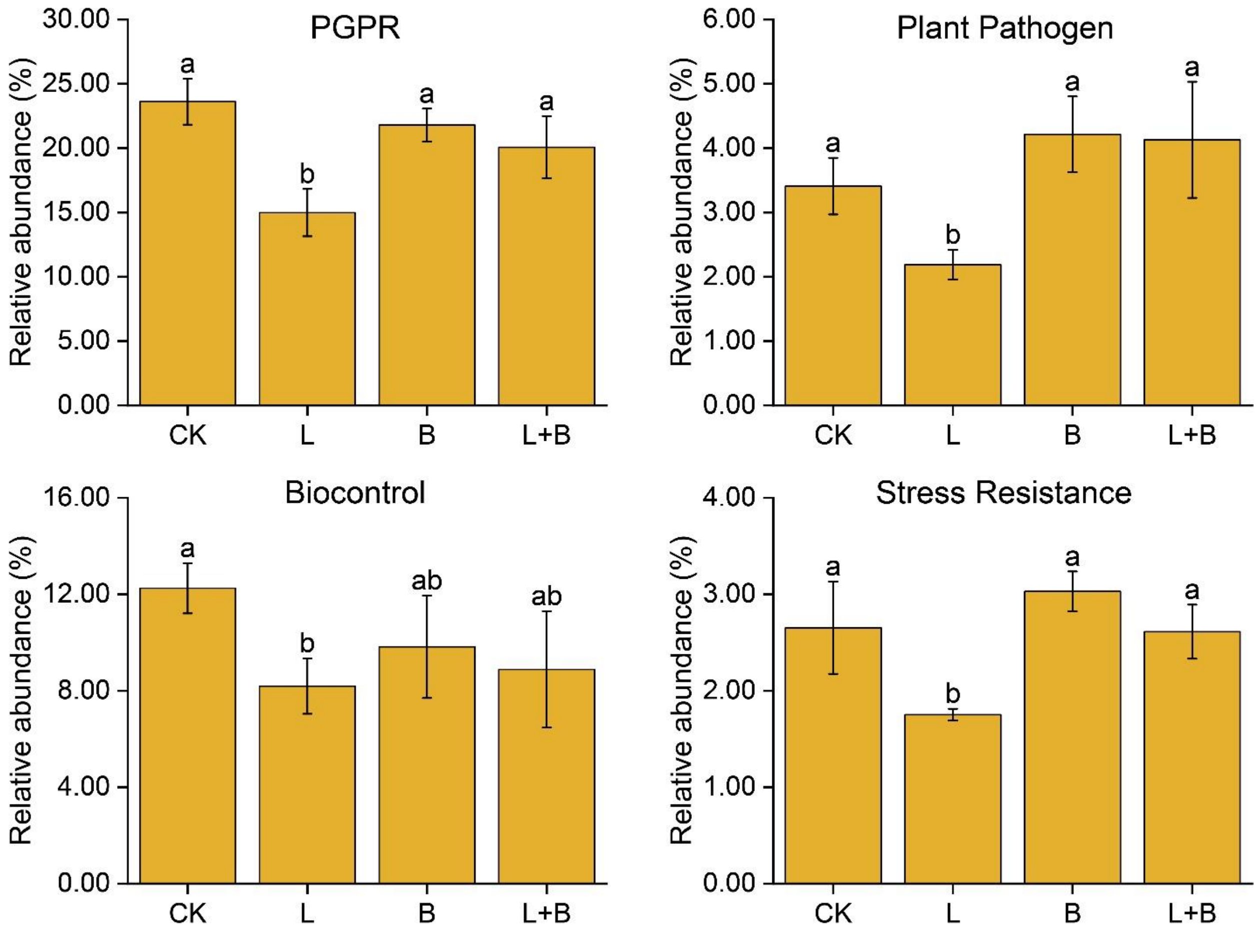

Fig.S2 Changes in the relative abundance of functional bacterial communities in the rhizosphere of tobacco. PGPR: plant growth promoting rhizobacteria. CK: control without soil amendment; L: lime treatment; B: biochar treatment; L+B: lime and biochar mixture treatment. Data depicts means ± SD of three biological replicates. Significant differences between treatments ($P < 0.05$) are illustrated by different lowercase letters
